# PVAT-conditioned media from Dahl S rats on high fat diet promotes inflammatory cytokine secretion by activated T cells prior to the development of hypertension

**DOI:** 10.1371/journal.pone.0302503

**Published:** 2024-10-03

**Authors:** Yining Jin, Sheng Liu, Kimberly E. Guzmán, Ramya K. Kumar, Luca M. Kaiser, Hannah Garver, Jamie J. Bernard, Sudin Bhattacharya, Gregory D. Fink, Stephanie W. Watts, Cheryl E. Rockwell

**Affiliations:** 1 Department of Pharmacology and Toxicology, College of Human Medicine, Michigan State University, East Lansing, MI, United States of America; 2 College of Osteopathic Medicine, Michigan State University, East Lansing, MI, United States of America; 3 Department of Medicine, College of Human Medicine, Michigan State University, East Lansing, MI, United States of America; 4 Department of Biomedical Engineering, College of Engineering, Michigan State University, East Lansing, MI, United States of America; Henan University, CHINA

## Abstract

There is considerable evidence that the immune system plays a role in hypertension, however this role is not fully characterized. Our previous studies demonstrated that mesenteric perivascular adipose tissue (mPVAT) harbors a large T cell population, which is a cell type identified as contributing to hypertension. In the present study, we tested the hypothesis that soluble mediators in mPVAT influence T cell function just prior to the development of hypertension. Toward this end, we utilized a unique model of hypertension in which Dahl S rats on a high fat (HF) diet develop hypertension. We found that conditioned media (CM) from mPVAT from healthy Dahl S rats on control diet buffers T cell activation, however, mPVAT-CM from Dahl S rats on a HF diet markedly increased inflammatory cytokine induction (IFNγ, GM-CSF and IL-17a) by activated T cells. These cytokines are known to promote activation of macrophages and neutrophils, among other effects. Conversely, the anti-inflammatory cytokine, IL-10, was not different between the groups, suggesting the effect is selective for inflammatory cytokines. Furthermore, we conducted bulk RNA-seq on activated T cells cultured in mPVAT-CM from Dahl S rats on either control (CTL) or HF diet for 10 weeks. In accordance with the cytokine analysis, mPVAT-CM from HF diet-fed rats significantly upregulated many genes associated with IFNγ/IL-17 induction, whereas Th2/Treg-associated genes were downregulated. Taken together, these data strongly suggest soluble mediators from mPVAT influence T cell inflammatory status and may promote Th1/Th17 differentiation preceding the development of hypertension triggered by HF diet.

## Introduction

High blood pressure (hypertension), traditionally defined as a systolic blood pressure greater than 130 mmHg or a diastolic blood pressure greater than 80 mmHg, is a primary cause for cardiovascular disease (CVD) mortality in the United States [1]. More than 47% of the adults in the U.S. have hypertension and most of them do not have their hypertension under control [1]. Additionally, resistant hypertension that does not respond well to the currently available treatments remain a significant problem [2]. Therefore, understanding the mechanisms that drive hypertension and exploring new therapeutic targets for treatment is vital.

Emerging evidence indicates that the immune system plays a causative role in hypertension. There is evidence to implicate a role for IFNγ producing CD8+ T cells, IL-17A producing CD4+ T cells and T regulatory cells in hypertension in different animal models [3–5]. Some of the strongest evidence comes from RAG1−/− Dahl S rats, RAG1−/− mice and SCID mice (all models lack both T and B cells), which show a diminished rise in blood pressure in experimental hypertension [6–9]. Immunosuppression was also reported to be protective against hypertension in a human study [10]. However, the source of immune cells that contribute to hypertension remains unclear.

Perivascular adipose tissue (PVAT), the fat around the peripheral blood vessels, has been identified as a key player in both cardiovascular homeostasis and disease [11–13]. Under physiological conditions, PVAT exerts anticontractile, anti-inflammatory and antioxidant effects [14–16]. On the contrary, in hypertension, PVAT is remodeled and loses its protective anti-contractile function [17–20]. Interestingly, as a tissue in close proximity to blood vessels, PVAT contains its own repertoire of immune cells, enriched compared to other adipose depots, which makes PVAT a potential source of immune cells involved in hypertension [21]. Our published data demonstrate that within the immune community hosted by PVAT, T cells and macrophages are the largest subpopulations [21]. Research from our group and others demonstrate that the interplay between adipocytes and immune cells in dysfunctional adipose tissue can clearly promote inflammation [22–26]. These studies and others support the hypothesis that inflammation contributes to the development of hypertension [27]. These previous findings prompted us to consider PVAT as a potential source of immune modulation during hypertension and lead us to investigate the influence of PVAT microenvironment on immune cell function during homeostasis and prior to the development of HF diet-induced hypertension.

In the current study, we activated T cells in the presence and absence of mesenteric PVAT-conditioned media (mPVAT-CM) from Dahl S rats on either a CTL or HF diet for 10 weeks, 17 weeks and 24 weeks. T cell activation status, effector function and gene expression were determined. We observed an increase in the induction of proinflammatory cytokines by activated T cells cultured in media conditioned with mPVAT taken from Dahl S rats on a HF diet. This effect was observed in mPVAT-CM taken from rats on HF diet for only 10 weeks, which precedes the development of hypertension in these animals (blood pressures were previously reported in [28]). In addition, we conducted RNA-sequencing on activated T cells cultured in mPVAT-CM. Genes associated with Th1/Th17 were found upregulated in the HF diet group. All these findings pinpoint the critical need to study the PVAT microenvironment and the role of T cells in PVAT during HF diet-induced hypertension.

## Materials and methods

### Animals

Dahl S rats (Charles River, Indianapolis, IN) were purchased at weaning and put on a CTL diet (10% Kcal fat and 0.2% salt) (Diet D12405J, Open Source) or HF diet (60% High fat as lard and 0.3% Na) (Diet D12492, Open Source). The rats were then maintained on the diet for the duration of the study. Data from this study was pooled from both male and female rats (no differences were noted between sexes). The male and female samples were roughly equal (3 male and 3 female for CTL group at 10 wk, 3 male and 2 female for HFD group at 10 wk, 4 male and 3 female for both groups at 17 wk, 5 male and 4 female for both groups at 24 wk). The blood pressures from these animals were previously reported [28]. Tissues were collected from animals following euthanasia by lethal overdose of pentobarbital in accordance with euthanasia methods approved by the American Veterinary Medical Association. Because the animals were not subjected to any procedures other than receiving special diets and blood pressure measurements, no other anesthetics or analgesics were needed or used during the course of this study. All animal experiments were conducted in accordance with the *Guide for Care and Use of Animals* as adopted by the National Institutes of Health and were approved by the Institutional Animal Care and Use Committee (IUCAC) at Michigan State University.

### mPVAT-CM preparation

The mPVAT was removed from the blood vessels, blotted dry, and weighed. The adipose tissue was transferred into a pre-chilled 5ml culture tube and received 500ul of Dulbecco’s Modified Eagle Medium (DMEM) (Genesee Scientific, El Cajon, CA). While the tube was on ice, a TissueRuptor (Qiagen, Germantown, MD) tip was inserted into the tube to homogenize the fat. Fat homogenate was transferred onto Millicell filter insert (Millipore Sigma, Burlington, MA). The filter inserts were loaded on a 6-well culture plate (Greiner Bio-One, Monroe, NC). The plate was incubated at 37°C for 10 minutes and then placed on a rotator shaker at 200rpm for one hour. Filtrate was collected in 1.5 mL centrifuge tubes. Protein concentration of the mPVAT-CM were determined by BCA Protein Assay Kit (ThermoFisher Scientific, Waltham, MA).

### Cell culture

Single cell suspensions from spleens of rats on either CTL or HFD were prepared in DMEM containing 10% fetal bovine serum (ThermoFisher Scientific, Waltham, MA), 25 mM HEPES (Sigma), 10 mM nonessential amino acids (Sigma), 100 ul/ml penicillin-streptomycin solution (Sigma), and 55 μM 2-mercaptoethanol (Invitrogen). The cells (2×10^6^ cells/ml) (100 ul/well) were cultured in an anti-rat CD3 pre-coated (5ug/mL, 200 ul/well) flat-bottom 96-well plate (Greiner Bio-One, Monroe, NC) in the presence of anti-rat CD28 (500 ug/mL, 1ul/well) with mPVAT-CM (36 mg/well) for incubation (37°C, 5% CO_2_). For each biological replicate, splenocytes were cultured in conditioned media from mPVAT derived from the same animal. There were no differences in cytokine production between splenocytes derived from rats on CTL vs HFD (in the absence of mPVAT-CM).

### ELISA

IL-2 levels of cell culture supernatants were measured using IL-2 Rat ELISA Kit (Invitrogen/ThermoFisher Scientific, Waltham, MA), according to the manufacturer’s instructions (sensitivity = 11 pg/ml) For certain studies, IL-17a was quantified using the Rat IL-17a Uncoated ELISA kit (Invitrogen/Thermofisher Scientific, Waltham, MA), according to the manufacturer’s instructions (sensitivity = 2 pg/ml).

### Multiplex assay

Cytokine levels of cell culture supernatants were determined using Milliplex kit (Millipore Sigma, Burlington, Massachusetts) and measured using Luminex 200 system (Luminex Corporate, Austin, TX). The assay sensitivity for the cytokines is: IL-17a and IL-10 (7.3 pg/ml), GM-CSF (12.2 pg/ml), IFNγ (14.6 pg/ml), TNFα (2.4 pg/ml), IL-4 (4.9 pg/ml)

### Flow cytometric analysis of T cells in the spleen

Single cell suspensions from the spleen were prepared in DMEM containing 10% fetal bovine serum. Viability was assessed using propidium iodide in a small fraction of cells (Biolegend, San Diego, CA). The rest of the cells were resuspended in FACS buffer and labeled with CD3-BV605 (BD Biosciences, Becton, NJ), CD4-AF647 (Biolegend, San Diego, CA) and CD8a-PerCP/eFluor 710 (ThermoFisher Scientific, Waltham, MA), CD25-PE (ThermoFisher Scientific, Waltham, MA), and CD134-(PE-Vio770) (Miltenyi Biotec, Gaithersburg, MD) for 30 min in the presence of anti-CD32 (BD Biosciences, Becton, NJ) and then washed. Fluorescence was detected by an Attune NxT flow cytometer (ThermoFisher Scientific, Waltham, MA).

### RNA isolation and bulk RNA sequencing

RNA from the cells was extracted using RNeasy Mini Kit (Qiagen, Germantown, MD). Transcriptome analysis of RNA sequencing (30M raw reads/sample) was performed via Illumina platforms by Novogene (Sacramento, CA). After quality control, HISAT2 was used to map the filtered sequenced reads to the reference genome. Gene expression level is estimated by the FPKM (Fragments Per Kilobase of transcript sequence per Millions base pairs sequenced) method. The coexpression Venn diagram was used to visualize the number of genes that are uniquely expressed within each group/sample. Differentially expressed genes (DEGs) analysis of HF condition and CTL condition was performed using the DESeq2 R package. Volcano plots was used to infer the overall distribution of DEGs. Hierarchical clustering analysis was carried out of log2(FPKM+1) of union DEGs, within all comparison groups.

### Statistical analysis

For comparison of two groups (CTL diet and HF diet), Student’s t-test was used (SigmaPlot 12 software). Data are shown as mean ± SEM. For all analyses, statistical significance level was set at p < 0.05.

### Ethics statement

All animal protocols are in compliance with the Guide for the Care and Use of Animals and were approved by the Institutional Animal Care and Use Committee (IACUC) at Michigan State University.

## Results

### Healthy mPVAT microenvironment buffers the T cell response to stimulation

**Fig 1:**
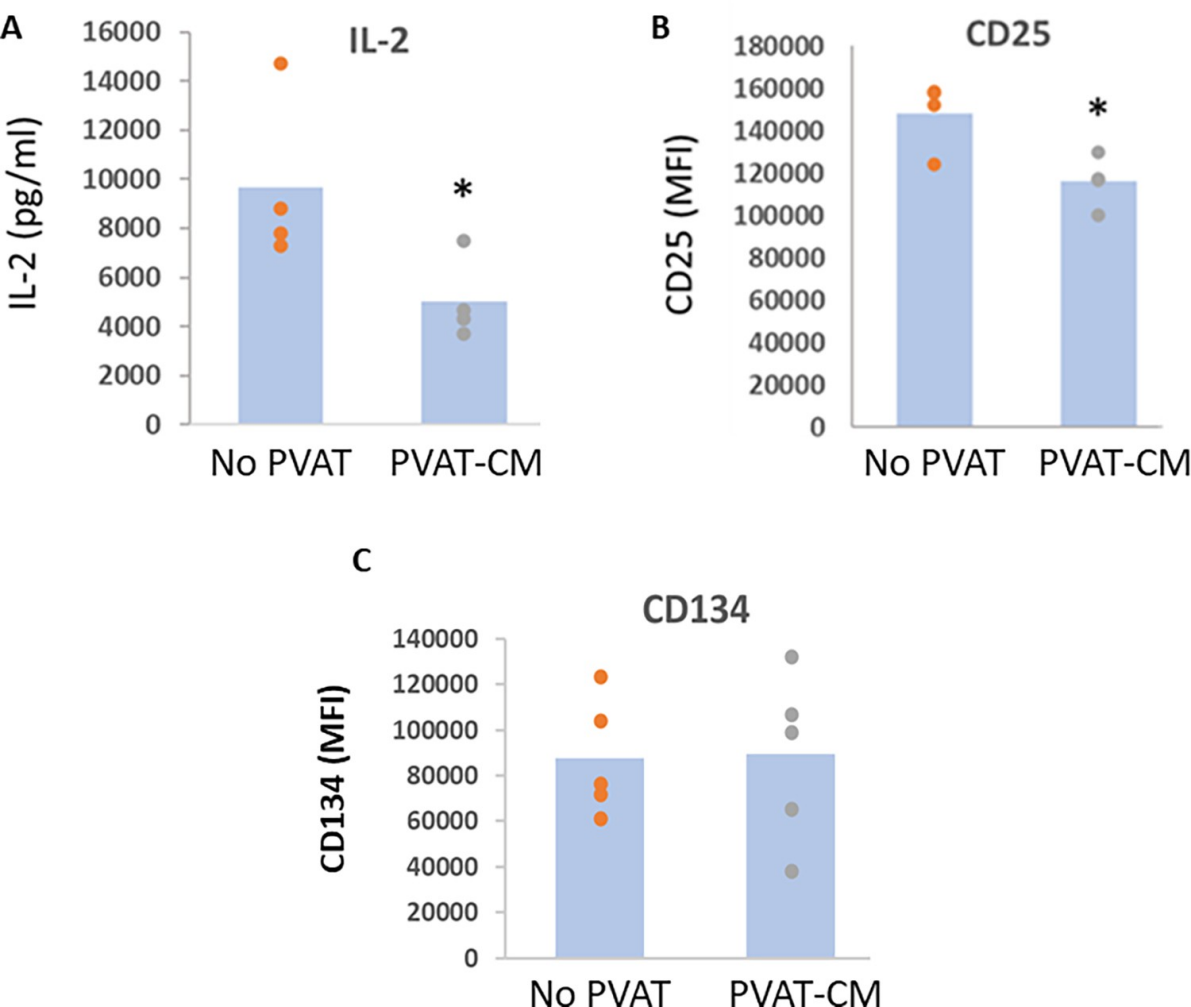
Healthy PVAT microenvironment buffers the T cell response to stimulation. Splenic T cells were cultured with mPVAT-CM from healthy rats on a normal control (CTL) diet for 10 weeks and stimulated with anti-CD3 and anti-CD28. Cells and supernatants were collected after 24 hours. (A) Under homeostatic conditions, PVAT-CM suppresses IL-2 secretion by activated T cells. IL-2 was quantified by ELISA from cell supernatants collected 24 h after T cell activation. (B) PVAT-CM modestly suppresses CD25 induction by activated T cells as quantified by flow cytometry (CD4+CD25+). The data are depicted as mean fluorescence intensity. (C) No significant difference in the expression of CD134 after treating T cells with mPVAT-CM. mPVAT and isolated splenocytes were collected from the same animal. The data are depicted as mean fluorescence intensity. *P<0.05 as compared to CTL diet. N = 4–5.

Splenic T cells were cultured with mPVAT-CM from healthy rats on a CTL diet for 10 weeks and stimulated with anti-CD3 and anti-CD28. Supernatants were collected after 24 hours (peak time-point for IL-2 secretion), and IL-2 was quantified by ELISA. Data show that mPVAT-CM from CTL animals suppressed the IL-2 induction in activated T cells by 50% compared to the group without CM (Fig 1A). In addition to IL-2 itself, a decrease in the induction of the high-affinity IL-2 receptor subunit CD25 was observed in the T cells cultured with mPVAT-CM (Fig 1B). To explore whether other early events following T cell activation were impacted, MFI of T cell costimulatory molecule OX-40 (CD134) was measured by flow cytometry. There was no significant difference found on the expression of CD134 after treating T cells with CM (Fig 1C). mPVAT-CM from healthy rats on CTL diet had no effect on production of IFNγ, GM-CSF, TNFα, IL-4 or IL-10 by splenic T cells 96 h after activation (S1 Fig).

### mPVAT-CM from HF diet skews immune cell function

**Fig 2:**
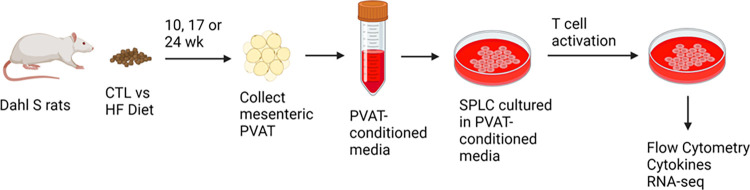
Schematic representation of experimental design. Dahl S rats were placed on a control (CTL) or high-fat diet (HFD) for 10, 17 or 24 wk after which spleens and mPVAT were collected. Conditioned media was prepared from the mPVAT as described in Methods. Splenocytes were treated with anti-CD3/anti-CD28 (a T cell specific activator) and cultured in PVAT-conditioned media for 48 or 96 h after which cell and cell supernatants were collected for analysis. Created with BioRender.com.

**Fig 3:**
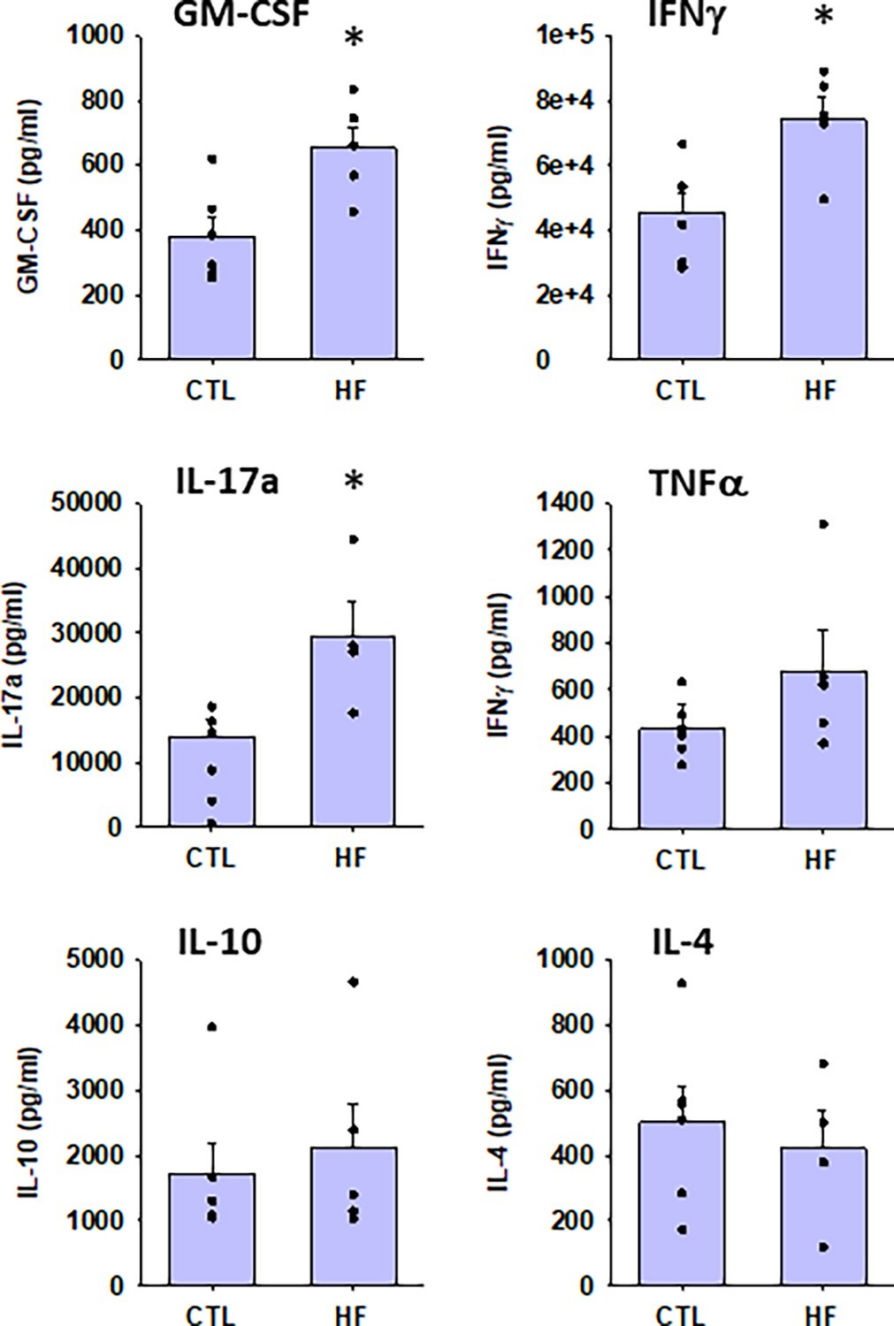
Conditioned media from mPVAT from rats on the HF diet for 10 wks greatly increased the induction of the pro-inflammatory cytokines secreted by activated T cells. Dahl S rats were placed on a control (CTL) or high-fat diet (HFD) for 10 wk after which spleens and mPVAT were collected. Conditioned media was prepared from the mPVAT as described in Methods. Splenocytes were treated with anti-CD3/anti-CD28 (a T cell specific activator) and cultured in PVAT-conditioned media for 96 h. Cytokines were quantified by multiplex bead assay (Luminex). mPVAT and isolated splenocytes were collected from the same animal. *P<0.05 as compared to CTL diet. N = 5 (CTL) and N = 6 (HFD).

**Fig 4:**
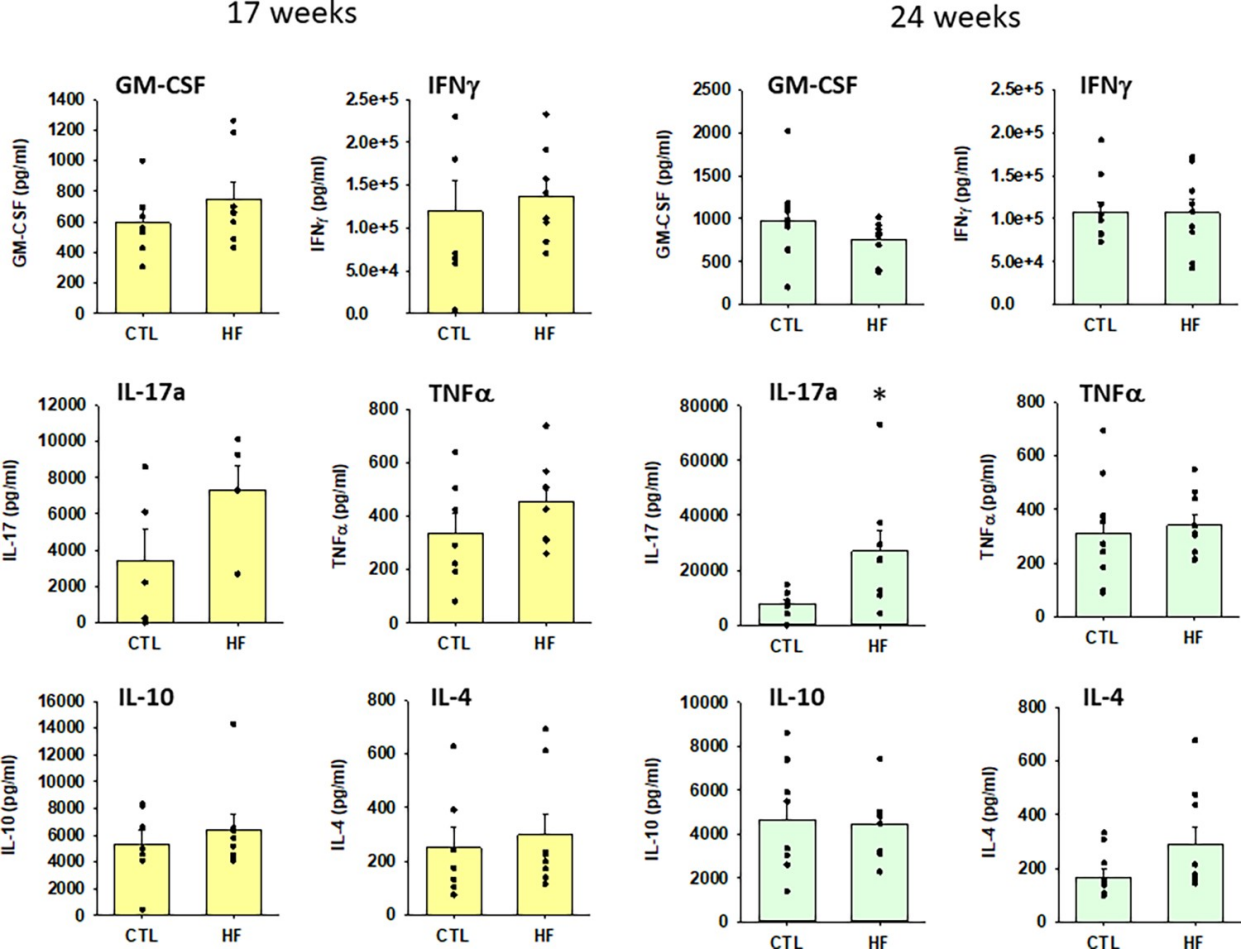
Conditioned media from mPVAT-CM from rats on the HF diet for 17 and 24 wks greatly increased the induction of IL-17a secreted by activated T cells. Dahl S rats were placed on a control (CTL) or high-fat diet (HFD) for 17 or 24 wk after which spleens and mPVAT were collected. Conditioned media was prepared from the mPVAT as described in Methods. Splenocytes were treated with anti-CD3/anti-CD28 (a T cell specific activator) and cultured in PVAT-conditioned media for 96 h. Cytokines were quantified by multiplex bead assay (Luminex). mPVAT and isolated splenocytes were collected from the same animal. *P<0.05 as compared to CTL diet. N = 7 (CTL and HFD at 17 wk) and N = 9 (CTL and HFD at 24 wk).

**Fig 5:**
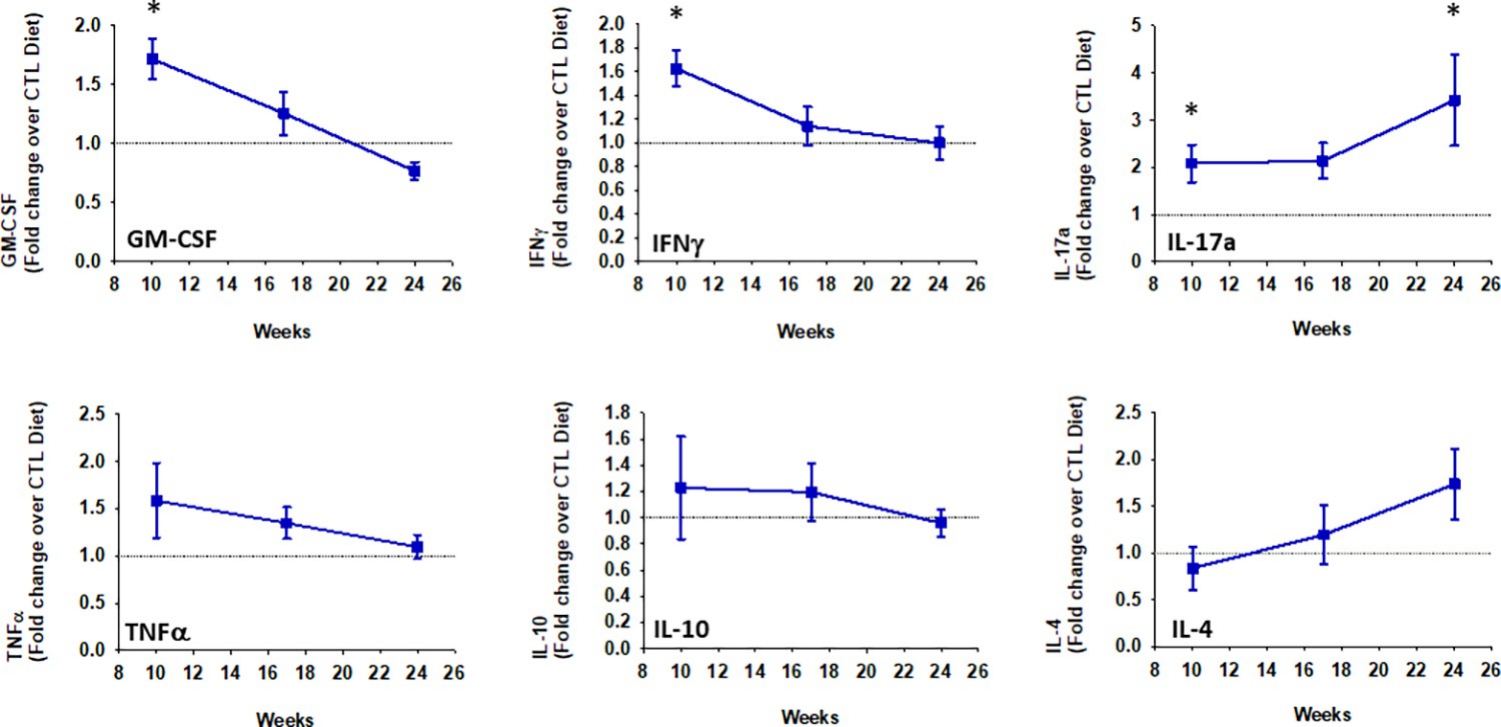
Dynamics of cytokines secreted by splenocytes cultured with mPVAT-CM from animals at each stage of hypertension. Dahl S rats were placed on a control (CTL) or high-fat diet (HFD) for 10, 17 or 24 wk after which spleens and mPVAT were collected. Conditioned media was prepared from the mPVAT as described in Methods. Splenocytes were treated with anti-CD3/anti-CD28 (a T cell specific activator) and cultured in PVAT-conditioned media for 96 h. Cytokines were quantified by multiplex bead assay (Luminex). mPVAT and isolated splenocytes were collected from the same animal. The data are presented as fold induction of cytokine induction after 10 wk, 17 wk and 24 wk of HF diet exposure over CTL diet.

Following the investigation of the effect of mPVAT on splenocytes from normotensive rats on CTL diet, the impact of mPVAT on immune cell function during HF diet-induced hypertension was examined before (10 wk) and during early (17 wk) and late (24 wk) hypertension (Fig 2, blood pressures from these animals were previously reported in [28]). Splenic T cells were cultured with mPVAT-CM from rats on either a CTL diet or HF diet for 10 wk and activated with anti-CD3 and anti-CD28 for 96 hours prior to cytokine analysis (96 h is the timepoint at which the late cytokines peak). Our data show that the induction of the pro-inflammatory cytokines, GM-CSF, IFNγ (7.5x10^4^ pg/ml) and IL-17a was greatly elevated in the HF diet group vs the CTL diet group (Fig 3). In contrast, there was no significant difference in the induction of IL-4 and IL-10 between the HF diet group and the CTL diet group (Fig 3). At the 10 wk timepoint, there was no significant difference in blood pressure [28]. In addition to the 10 wk timepoint, we also investigated the impact of mPVAT-CM from rats on diet for either 17 or 24 wk at which time the rats in the HF diet group are hypertensive [28]. GM-CSF and IFNγ levels declined after 17 weeks of HF diet and were no longer significantly elevated compared to CTL diet (Figs 4 and 5). In contrast, IL-17a showed no recovery and was consistently elevated by mPVAT-CM from HF diet-fed rats for 24 weeks (Figs 4 and 5). There were no differences in IL-4 and IL-10 between the diet groups at any time point.

### Flow cytometric analysis of splenocytes cultured with mPVAT-CM

**Fig 6:**
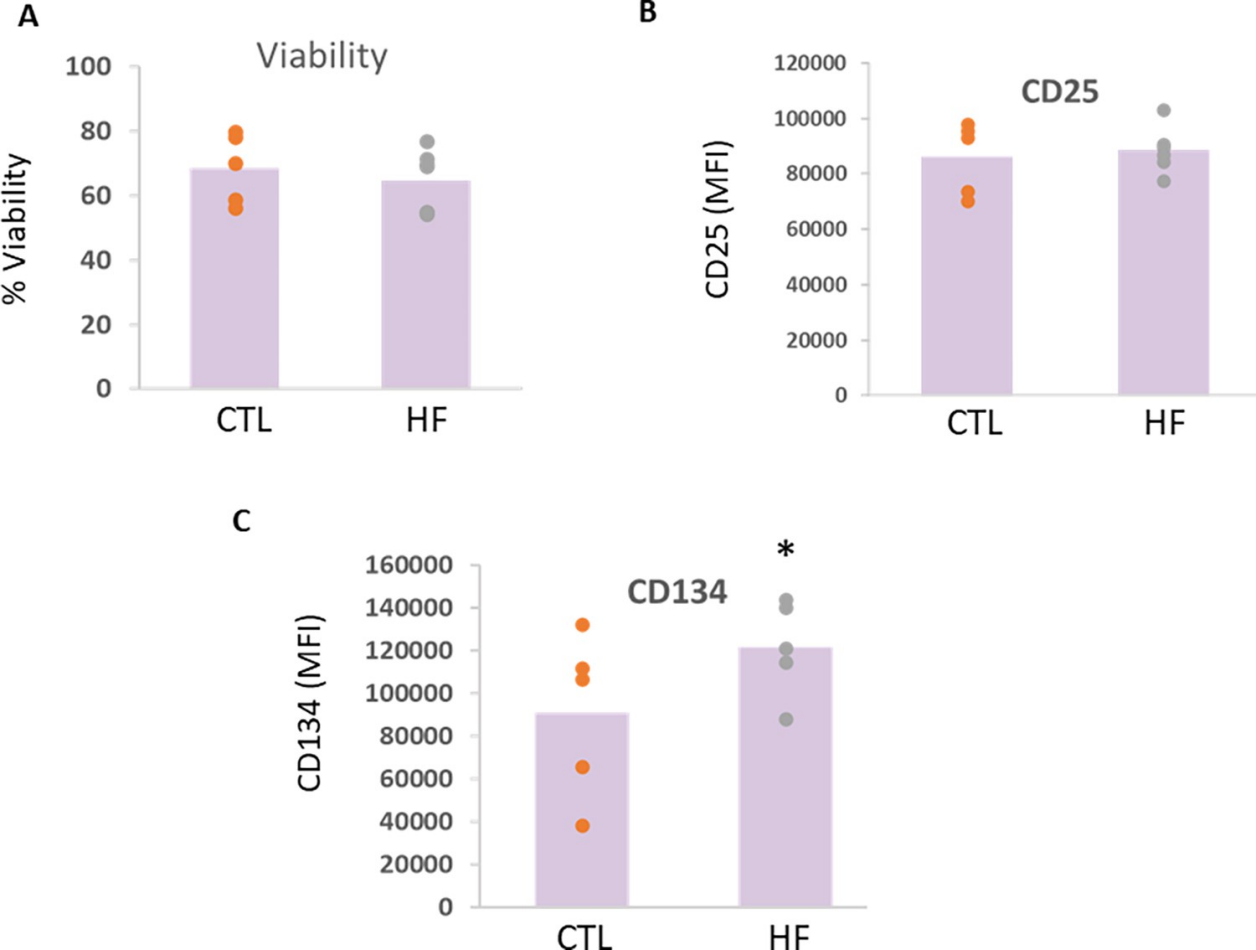
Flow cytometric analysis of splenocytes cultured with mPVAT-CM. Dahl S rats were placed on a control (CTL) or high-fat diet (HFD) for 10 wk after which spleens and mPVAT were collected. Conditioned media was prepared from the mPVAT as described in Methods. Splenocytes were treated with anti-CD3/anti-CD28 (a T cell specific activator) and cultured in PVAT-conditioned media for 48 h. (A) Cell viability was determined by propidium iodide staining and flow cytometry. (B-C) MFI of (B) theT cell high affinity IL-2 receptor CD25 and (C) the T cell costimulatory molecule CD134 was measured by flow cytometry. *P<0.05 as compared to CTL diet. N = 4–5.

Flow cytometric analysis was performed on splenic T cells from CTL group, cultured with mPVAT-CM from rats on either a CTL diet or HF diet for 10 weeks. Cell viability was not different between the groups as determined by propidium iodide staining (Fig 6A). T cell high affinity IL-2 receptor CD25 and T cell costimulatory molecule CD134 were measured. While no differences in CD25 expression were observed, expression of the costimulatory molecule CD134 was significantly increased in splenocytes cultured with mPVAT-CM from rats on HF diet (Fig 6).

### Bulk RNA-sequencing analysis of activated T cells cultured with mPVAT-CM

**Fig 7:**
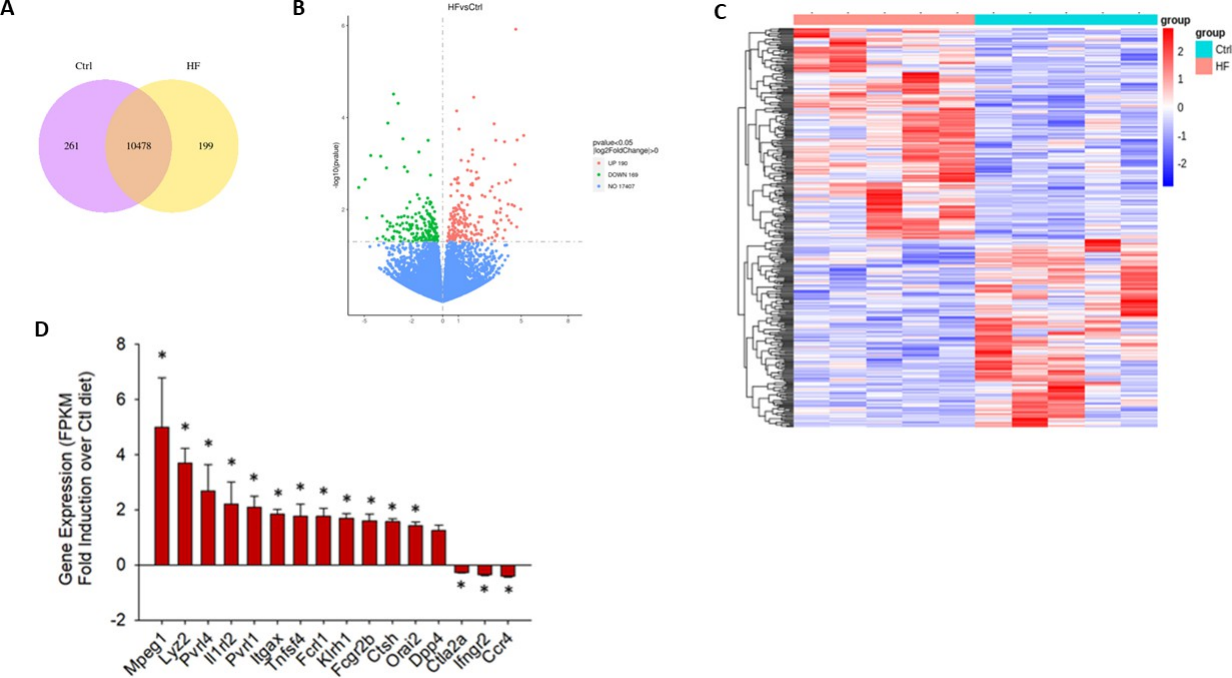
Bulk RNA-sequencing analysis of activated T cells cultured with mPVAT-CM. (A) The number of genes expressed in HF and CTL diet group was presented in the Coexpression Venn Diagram. (B) Analysis of DEGs from T cells cultured in PVAT-CM from rats on a HF diet as compared to CTL diet. (C) Cluster analysis was performed on DEGs. (D) Differentially expressed immunomodulatory genes are depicted as fold change over CTL (FPKM). *p<0.05 as compared to CTL diet. N = 5.

To identify genes that impact immune cell fate and function, a bulk RNA-sequencing approach was conducted on activated T cells cultured in mPVAT-CM from rats on either CTL or HF diet for 10 weeks and activated with anti-CD3 and anti-CD28 for 96 hours. The level of gene expression was analyzed, and the number of genes that are uniquely expressed within the HF group and CTL diet groups, as well as the overlapping regions showing the number of genes that are co-expressed in two groups, is represented by the Coexpression Venn Diagram (Fig 7A). Among the differentially expressed genes (DEGs), T cells cultured in mPVAT-CM from rats on a HF diet had 190 upregulated genes and 169 downregulated genes as compared to T cells cultured in mPVAT-CM from rats on CTL diet (Fig 7B). To analyze and visualize differential gene expression patterns, a heatmap cluster analysis was performed on DEGs. Distinctive trends in expression patterns were observed between HF diet and CTL diet groups (Fig 7C).

### mPVAT-CM from HF diet-fed rats induced genes in T cells associated with activation, IFNγ and IL-17a signaling pathways

Within the DEGs set, we detected many immune-related genes. Genes associated with IFNγ/IL17a downstream effector function such as Mpeg1, Lyz2, Tnfsf4 (OX-40L) and Cathepsin H (Ctsh) were upregulated in the HF group, as shown in (Fig 7) and Table 1. In addition to these genes, other IFNg target genes such as Itgax (CD11c) and Fcgr2b were also upregulated. Consistent with this, the Ifngr2 gene encoding the beta chain of the interferon gamma receptor, which inhibits Th1 differentiation and function, was downregulated in the HF group. Likewise, the Th2-associated chemokine receptor, Ccr4 was downregulated in the HF group. Ctla-2, a protease that enables bystander CD4+ T cells to be converted to Treg cells by TGFβ promotion, was also downregulated, consistent with increased production of IFNγ and IL-17a. In addition, we also observed increased expression of genes associated with T cell activation, including Orai1 and Tnfsf4 (promote activation) and Il1r2 (induced in activated T cells).

**Table 1:** Gene expression shown as fold induction over CTL diet as determined by bulk RNA-seq.

	Genes associated with Th1/Th17			Genes associated with Th2/Treg	
Gene	Mpeg1	Lyz2	Tnfsf4	Ctla2a	Ccr4
Expression Fold over CTL	5.0±1.78	3.70±0.53	1.78±0.42	-0.27±0.02	-0.41±0.03

## Discussion

Our HF-diet induced hypertension rat model was previously described in Fernandes et al., in which Dahl S rats were put on HF diet from weaning to induce hypertension [29]. Using this model, we previously reported that after 10 weeks on the HF diet, blood pressure did not differ from rats on CTL diet in this study [28]. At 17 weeks, blood pressure in HF diet rats was significantly higher than in the CTL diet group and continued to be further elevated after 24 weeks [28]. We previously demonstrated that PVAT hosts a robust immune community, with T cells and macrophages being the largest contributors in number and function [28]. Other groups demonstrated that immune cells within visceral adipose tissue play important roles under physiological conditions, including tissue housekeeping, immunometabolism and maintenance of tissue homeostasis [30–32]. However, under pathological conditions, visceral adipose tissue is dysfunctionally remodeled. This leads to changes in immune function, such as an increase in the number and activation of macrophages and Th1 cells, while simultaneously reducing others cell types, including eosinophils, Th2 cells and Tregs [30, 31, 33, 34]. The shift in immune cell phenotypes, numbers and secreted cytokines impair immune homeostasis within adipose tissue and result in local and systemic inflammation. Given that numerous studies demonstrate a role for T cell-driven inflammation in vascular dysfunction in combination with the proximity of PVAT T cells to the vasculature, the goal of this study was to determine the impact of the PVAT microenvironment on T cell function [35, 36].

In this study, we explored the influence of the PVAT microenvironment from CTL rats on immune cell function during homeostasis. We found that PVAT tempers the T cell response to stimulus through suppression of growth factor IL-2 and the IL-2 high affinity receptor CD25 but did not impact other T cell pathways such as T cell costimulatory molecule CD134 or other cytokines. We then determined the effector function of T cells impacted by the PVAT microenvironment prior to and during the development of high fat diet-induced hypertension. Our data suggest that PVAT promotes a pro-inflammatory phenotype in T cells prior to HF diet-induced hypertension. Pro-inflammatory cytokines secreted by activated T cells such as GM-CSF, IFNγ and IL-17a were significantly elevated in immune cells cultured in PVAT-CM from animals on HF diet for 10 weeks. The rise in the cytokines GM-CSF, IFNγ and IL-17a would be expected to potentiate pro-inflammatory signaling by recruiting and priming immune cells such as macrophages, neutrophils, NK cells and Th1 cells. Interestingly, the dynamics of these cytokines were different at each stage of hypertension. For example, IL-17a was consistently elevated in cells cultured in PVAT-CM from rats on the HF diet for 17 weeks and 24 weeks. However, GM-CSF and IFNγ were increased in cells cultured in PVAT-CM from rats on HF diet for 10 weeks but recovered to normal levels in PVAT-CM from rats after 17 weeks of HF diet. Notably, there was no compensatory increase in the anti-inflammatory cytokine IL-10 at any time point, suggesting that protective regulatory countermeasures were not deployed prior to or during the development of hypertension in this model. Likewise, there was no difference in the level of IL-4 (a cytokine that suppresses IFNγ and IL-17a induction) between the HF diet and the CTL diet group at all time points. Overall, these results suggest that the mPVAT microenvironment, while protective in healthy animals, switches to an inflammation-promoting phenotype in the context of HF diet; this change occurs prior to the development of HF diet-induced hypertension.

In addition to differences in cell function, bulk RNA-sequencing conducted on activated T cells cultured in PVAT-CM revealed distinctive trends in expression patterns between the HF diet and the CTL diet group. Like the pro-inflammatory cell function phenotype found in the HF diet group, genes associated with pro-inflammatory T cell subsets (Th1, Th17, etc.) such as Mpeg1, Lyz2, and Ctsh were upregulated, whereas Th2/Treg related genes such as Ccr4 and Ctla-2 were downregulated in the HF diet group. Collectively, the RNA-sequencing data indicated that the shift toward a pro-inflammatory phenotype observed at the functional level was also found on the RNA level in T cells cultured in PVAT-CM from the HF diet group. Interestingly, RNA-sequencing did not show differences in the mRNA expression of transcription factors that regulate T cell polarization (T-bet, GATA-3, RORγt and Foxp3). While the reason for this is unclear, it may be due to reduced sensitivity of RNA-seq (at moderate read coverage), the relatively low abundance of subpopulations expressing these genes, or differences in the kinetics of mRNA induction relative to cytokine secretion.

Factors that impact T cell polarization often have diametrically-opposed effects on different T cell lineages. For example, IL-4 promotes Th2 polarization and suppresses Th1 polarization. While our data show an increase in the production of GM-CSF, IFNγ and IL-17a by activated T cells cultured in mPVAT-CM from rats on high fat diet, we did not observe a correlative decrease in the Th2 cytokine, IL-4. This may indicate that PVAT-CM from rats on HFD may be somewhat specific to Th1/Th17 cytokines and does not have a direct effect on Th2 cytokine production per se. Interestingly, however, we observed a decrease in some of the Th2-associated genes from the RNA-sequencing. At this point, the reason behind the discordance in these observations is not clear. However, if mPVAT-CM promotes Th1/Th17 cytokine production without having direct effects on Th2 cytokines per se, the decrease in Th2-associated genes may have occurred as a result of high IFNγ/IL-17a levels rather than the conditioned media itself. It would be interesting to determine whether the increased IFNγ/IL-17a levels might start to impact Th2 cytokine production over time. It should be noted that while we did not see a decrease in IL-10, a regulatory T cell (Treg)-associated cytokine, IL-10 is a relative non-specific marker for Treg’s however. While there is a strong correlation of IL-4 with type 2 immune responses, in particular as a product of Th2 cells, IL-10 is an anti-inflammatory cytokine produced by many different cell types, including macrophages, Th2 cells and even Th1 cells.

We used mPVAT- conditioned media in these studies to understand the effect of the mPVAT microenvironment on cytokine production by activated T cells. This approach allowed us to maintain the influence of the mPVAT microenvironment during T cell activation and differentiation. However, we also recognize the benefits of directly characterizing the T cells found in mPVAT itself. While there are advantages to this approach, it will also be challenging due to the small size of mPVAT which yields limited numbers of immune cells. A single cell transcriptomics analysis (a method that works well with limited cell number) would allow for the characterization of immune cells in PVAT at least at the gene expression level and will be the focus of future studies.

Over the past 15 years, the hypertension community has returned to an idea introduced in the 1960s, that the immune system plays a role in hypertension. Specifically, studies support that immunosuppression protects against hypertension in humans and animals, suggesting an activated immune system plays a causative role in hypertension [10, 37–39]. T cells, in particular, have been identified has causing or contributing to hypertension in a number of models of hypertension. Guzik et al. demonstrated Ang II-induced hypertension was ablated in Rag1-/- mice (which lack T cell and B cells); the effect could be rescued by transferring wild-type T cells into the mice [8]. The Mattson lab demonstrated that the development of hypertension in Dahl S rats on a high salt diet was attenuated in Rag1 -/- Dahl S rats (lacking T and B cells) and CD247 -/- Dahl S rats (lacking functional T cells) [6]. SCID mice (a different mouse strain which lack B and T cells) have a blunted hypertensive response to angiotensin II [7]. These studies, in combination with the earlier T cell depletion studies from the twentieth century, provide strong evidence for a role for T cells in hypertension induced by a number of different stimuli. Although the results from the studies in Rag1-/- mice could not be repeated several years later (the discrepancy is thought to be due to genetic drift in the Rag1-/- strain), on the whole, there is still considerable evidence for a role of T cells in the development of hypertension. While the current study did not focus on the role of T cells in hypertension, our data indicate that the PVAT microenvironment has the capacity to promote a pro-inflammatory status in T cells in Dahl S rats on a high fat diet prior to the development of hypertension in this model.

### Limitations

We acknowledge limitations to this work. We pooled data from both male rats and female rats in this study due to a limited sample size. Although Fernandes et al., reported there was no difference observed in elevated blood pressure between male and female Dahl S rats on HF diet, we can not exclude the potential for sex differences in cytokine production and immune cell gene expression in this model [29]. While we determined that HF diet impacts the PVAT microenvironment with marked functional changes on immune cell phenotype, the identification of the soluble mediators within PVAT-CM that cause this effect was beyond the scope of the present study. To address this in the future, lipidomic and proteomic studies would be useful in identifying potential mediators. Lastly, this study did not evaluate the impact of the PVAT microenvironment on the function of other immune cell types such as macrophages, neutrophils and NK cells, but we hope to investigate this in future studies.

Taken together, the results of this study suggest that the mPVAT microenvironment, while protective in healthy animals, switches to an inflammation-promoting phenotype in the context of HF diet, and this change occurs prior to the development of HF diet-induced hypertension.

## Supporting information

S1 Fig In healthy normotensive animals, PVAT-conditioned media does not impact induction of GM-CSF, IFNg, TNFa, IL-4 or IL-10. Conditioned media was prepared from the mPVAT as described in Methods. Splenocytes were treated with anti-CD3/anti-CD28 (a T cell specific activator) and cultured in PVAT-conditioned media for 96 h. Cytokines were quantified by ELISA (IL-17a) or multiplex bead assay (Luminex for all other cytokines). mPVAT and isolated splenocytes were collected from the same animal. *P<0.05 as compared to CTL diet.
(TIF)

S2 Fig High fat diet does not impact cytokine induction by splenocytes in the absence of PVAT-conditioned media. Splenocytes were isolated from Dahl S rats on either a control (CTL) or high-fat diet (HFD). Splenocytes were treated with anti-CD3/anti-CD28 (a T cell specific activator) and cultured for 96 h. Cytokines were quantified by ELISA (IL-17a) or multiplex bead assay (Luminex for all other cytokines). There were no statistical differences between the groups (at p < 0.05). N = 9.
(TIF)
