# Supplementary figures and images for: PVAT-conditioned media from Dahl S rats on high fat diet promotes inflammatory cytokine secretion by activated T cells prior to the development of hypertension
Source: PLoS One. 2024 Oct 3;19(10):e0302503. doi: 10.1371/journal.pone.0302503 (PMC11449284; doi:10.1371/journal.pone.0302503)

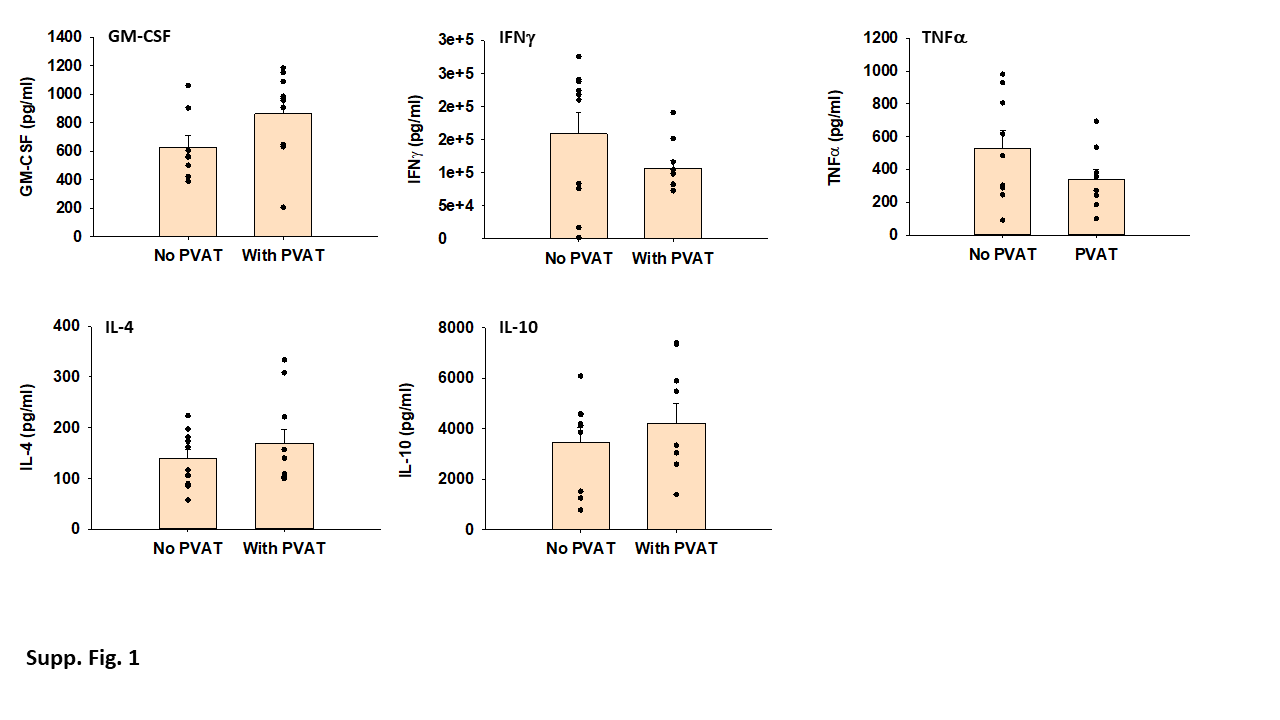

Supplement: S1 Fig — Conditioned media was prepared from the mPVAT as described in Methods. Splenocytes were treated with anti-CD3/anti-CD28 (a T cell specific activator) and cultured in PVAT-conditioned media for 96 h. Cytokines were quantified by ELISA (IL-17a) or multiplex bead assay (Luminex for all other cytokines). mPVAT and isolated splenocytes were collected from the same animal. *P<0.05 as compared to CTL diet. (TIF) [file pone.0302503.s001.tif]

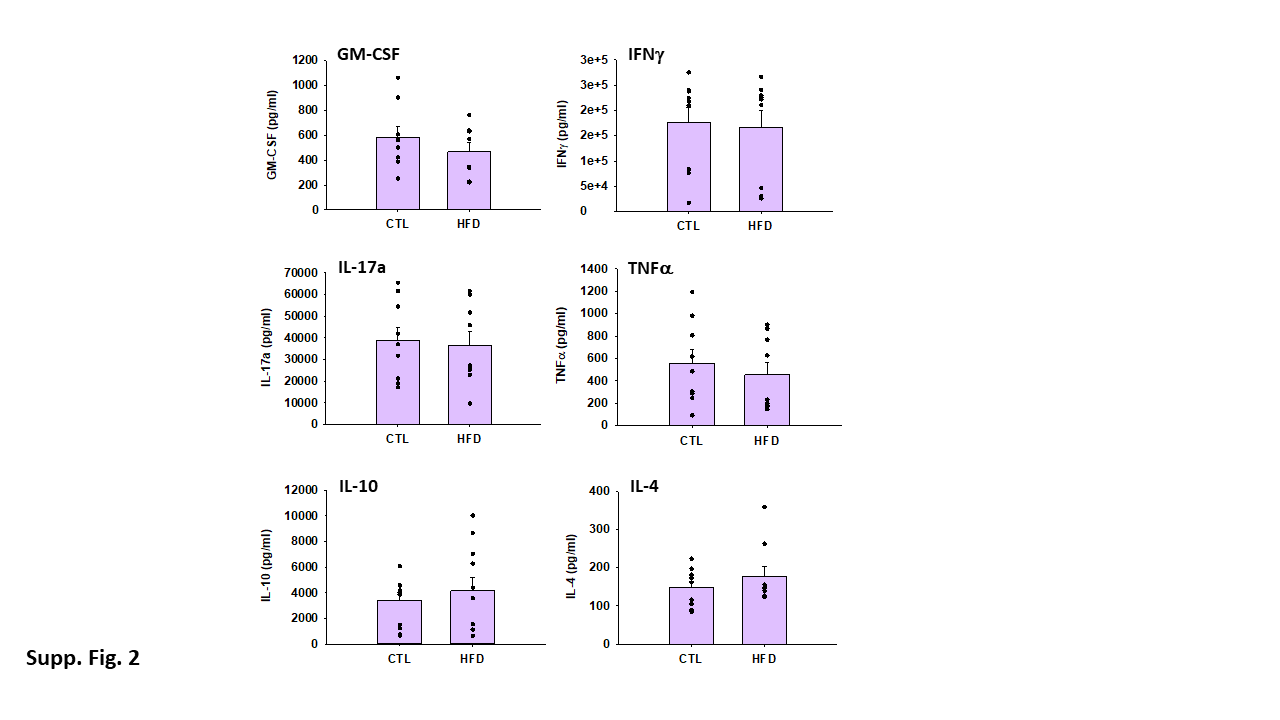

Supplement: S2 Fig — Splenocytes were isolated from Dahl S rats on either a control (CTL) or high-fat diet (HFD). Splenocytes were treated with anti-CD3/anti-CD28 (a T cell specific activator) and cultured for 96 h. Cytokines were quantified by ELISA (IL-17a) or multiplex bead assay (Luminex for all other cytokines). There were no statistical differences between the groups (at p < 0.05). N = 9. (TIF) [file pone.0302503.s002.tif]
